# ﻿*Tarennapendula* (Rubiaceae), a new species from Guangxi, China

**DOI:** 10.3897/phytokeys.257.148816

**Published:** 2025-06-03

**Authors:** Yong-Hua Qin, Rainer W. Bussmann, Zhi-Rong Liu, Man Li, Chang-Ying Xia, Wang-Hui Wu, Sheng-Xiang Yu

**Affiliations:** 1 Guangxi Forestry Inventory and Planning Institute, CN-530011, Nanning, Guangxi, China Guangxi Forestry Inventory and Planning Institute Nanning China; 2 Institute of Botany and Bakuriani Alpine Botanical Garden, Ilia State University, Botanical Str. 1, 0105 Tbilisi, Georgia Ilia State University Tbilisi Georgia; 3 Department of Botany, State Museum for Natural History Karlsruhe, Erbprinzenstraße 13, 76133 Karlsruhe, Germany Department of Botany, State Museum for Natural History Karlsruhe Karlsruhe Germany; 4 Guangxi Huasen Design Consulting Co., Ltd., CN-530011, Nanning, Guangxi, China Guangxi Huasen Design Consulting Co., Ltd. Nanning China; 5 College of Teacher Education, Southwest University, CN-400715, Chongqing, China Southwest University Chongqing China; 6 State Key Laboratory of Plant Diversity and Specialty Crops, Institute of Botany, Chinese Academy of Sciences, Beijing 100093, China Institute of Botany, Chinese Academy of Sciences Beijing China; 7 China National Botanical Garden, Beijing 100093, China China National Botanical Garden Beijing China; 8 College of Life Sciences, University of Chinese Academy of Sciences, Beijing 100049, China University of Chinese Academy of Sciences Beijing China

**Keywords:** Distribution, Ixoroideae, morphology, Pavetteae, taxonomy

## Abstract

The species *Tarennapendula* (Rubiaceae), a small shrub, is newly described and illustrated from southwestern Guangxi Autonomous Region, China. This species is similar to *Tarennatsangii* but is readily distinguished by its asymmetrical leaves, larger vegetative leaves than inflorescence leaves, adaxially pilosulous blades, pendulous inflorescences, longer pedicels (15–35 mm vs. 4–7 mm in *T.tsangii*), shorter corolla tubes (1–1.4 cm vs. 1.8–1.9 cm), multiple ovules per locule (vs. two), and sparsely pubescent fruits containing 13–19 seeds (vs. glabrous fruit containing only four seeds).

## ﻿Introduction

*Tarenna* Gaertn. is among the largest genera of the tribe Pavetteae, consisting of ca. 200 species within continental Africa, Madagascar, western Indian Ocean islands, Asia, and the Pacific region (De Block et al. 2015). The genus is distributed in primary evergreen forests and scrubs, in lowlands as well as at higher elevations. Recently, the circumscription of *Tarenna* was amended by the transfer of all African, Madagascan, and western Indian Ocean island species possessing fruits with a single, usually ruminate, seed to *Coptosperma* (Degreef et al. 2001; De Block et al. 2001). Even in this narrower sense, *Tarenna* remains variable in certain morphological characters, such as flower, fruit, seed, and pollen types (De Block and Robbrecht 1998; De Block et al. 2001), which has raised questions as to its delimitation (Bridson 1979; Smith and Darwin 1988; De Block et al. 2001, 2015). Therefore, further studies on the plant diversity and phylogeny of *Tarenna* are needed.

During field expeditions in southern China, which is a major biodiversity hotspot (Myers et al. 2000; Hou et al. 2010) distinguished by extraordinarily high vascular plant species diversity and endemism (Xu 1995; Peng et al. 2014), a distinct *Tarenna* specimen was discovered in the Defu Nature Reserve in Napo, Guangxi Autonomous Region. A critical comparison of specimens (PE, IBK, and KUN) and literature (Chen and Taylor 2011) demonstrated that this newly discovered specimen represented a new species of *Tarenna*, which we describe and illustrate as *Tarennapendula* sp. nov.

## ﻿Taxonomy

### ﻿ Tarenna pendula

Taxon classificationPlantaeGentianalesRubiaceae

Y.H.Qin, S.X.Yu & W.H.Wu sp. nov.

490566F8-EFB6-5F61-991B-5D21C3716FCB

urn:lsid:ipni.org:names:77361544-1

Figs 1 , 2 , 3

#### Diagnosis.

Similar to *Tarennatsangii*, but distinguished by its small form, asymmetrical leaves, vegetative leaves larger than inflorescence leaves, blades adaxially pilosulous, pendulous inflorescences, longer pedicels (15–35 mm vs. 4–7 mm in *T.tsangii*), shorter corolla tubes (1–1.4 cm vs. 1.8–1.9 cm), multiple ovules per locule (vs. 2), and sparsely pubescent fruits with 13–19 seeds (vs. glabrous fruit containing only 4 seeds).

#### Type.

China • Guangxi Autonomous Region: Baise City, Napo County, Defu Nature Reserve, bamboo understory on Baxiong Mountain, 1380 m a.s.l., 23.292437°N, 105.784845°E, 8 July 2021, *Yonghua Qin YH2021075* (holotype: PE, isotype: IBK).

#### Paratypes.

China • Guangxi Autonomous Region: Baise City, Napo County, Defu Nature Reserve, bamboo understory in evergreen broadleaf forest on southern slope of Baxiong Mountain, 1380 m a.s.l., 23.292451°N, 105.784899°E, 30 May 2023, *Yonghua Qin YH2023069* (PE, IBK); • Baise City, Napo County, Defu Nature Reserve, bamboo understory in evergreen broadleaf forest on the southern slope of Baxiong Mountain, 1380 m a.s.l., 23.292485°N, 105.7847944°E, 7 September 2023, *Yonghua Qin YH2023116* (PE, IBK).

**Figure 1.**
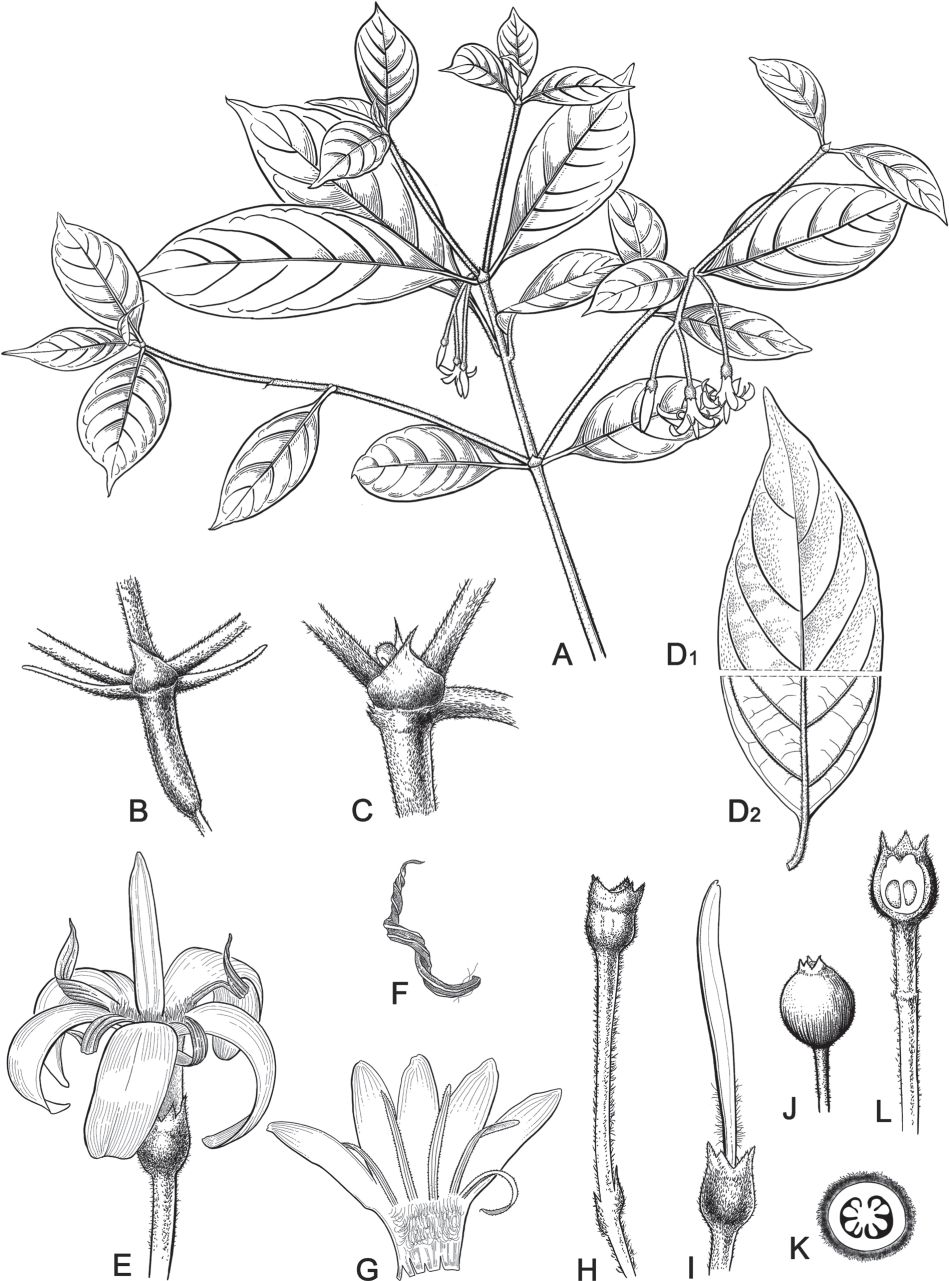
*Tarennapendula* sp. nov. **A** flowering branches **B** portion of inflorescence showing stipule **C** portion of stem showing stipule **D** portions of adaxial and abaxial leaf surfaces, (**D1**) pressure side of blade, (**D2**) low pressure side of blade **E** flower **F** stamen **G** longitudinally opened corolla showing the position of the stamens **H** pedicel, bracteoles, ovary and calyx **I** ovary, calyx, style and stigma **J** fruit **K** cross section of ovary **L** longitudinal section of fruit. Drawings by Lijie Zhu, based on type specimens.

#### Description.

Erect shrub up to 1–2 m tall, with a single main stem; branches flattened, shortly pilosulous, first year branchlets green, becoming brown or grayish-brown with age. Leaves opposite; petiole ca. 1 cm long, pilosulous; leaf blades asymmetrical, papery and blackish-brown when dried, oblong–obovate or oblanceolate, 4–17 cm × 1.5–4 cm, vegetative leaves large, 7–17 cm × 3–4 cm, inflorescence-supporting leaf pairs small, 4–7 cm × 1.5–3.5 cm, adaxially with adnate short hairs, abaxially scabrous and sparsely puberulent to subglabrous, with pubescence denser along principal veins, base cuneate, apex acuminate or shortly acuminate; midrib flat on upper surface, prominent on lower surface; secondary veins in 5–7 pairs, prominent on lower surface; stipules triangular, 3–5 mm, acuminate or apiculate when longer, persistent. Inflorescence corymbose, axillary or sometimes terminal, pendulous, 5–7 cm × ca. 3 cm, 3-5- or sometimes 9-flowered, shortly pedunculate, peduncles 3–8 mm, inflorescence axes and pedicels gray strigillose. Rachis unbranched. Flowers opposite on rachis, pedicels 1.5–3.5 cm long, proximal pedicels long, 2.5–3 cm long, distal pedicels short, 1.5–2 cm long. Calyx green, pilosulous; hypanthium portion cylindrical–urceolate, ca. 1 mm long; limb ca. 0.5 mm long, lobed for up to 1/2 of length. Corolla white, 1.5–1.9 cm long; tube cylindrical, ca. 5 mm long, inside at the upper part near the throat with dense hairs and at the lower part glabrous or sparsely pubescent, outside glabrous; lobes 5, contorted to the left in bud, oblong, 10–14 × 2–3 mm, with rounded apex, adaxial surface glabrous or sparsely pubescent at base, abaxial surface glabrous. Stamens inserted in corolla throat, alternating with corolla lobes, attached to the throat by a short filament; anthers yellowish-gray, linear, 8–10 mm long, basifixed, twisted after spreading. Style-stigma complex 1–1.2 cm long, exserted for 5–7 mm from corolla tube at anthesis; style sparsely pubescent at the middle; stigma fusiform, acentric apices. Ovary small, usually cup-shaped, 0.5–1 mm long, with 2 locules, each axile placenta with multiple ovules. Fruits berry-like, subglobose, 5–6 mm in diameter, with short persistent calyx teeth or calyx scar at the apex; seeds 13–19 per fruit, elliptic, 3–3.2 × 1.8–2 mm, brownish or black, testa rugose.

**Figure 2.**
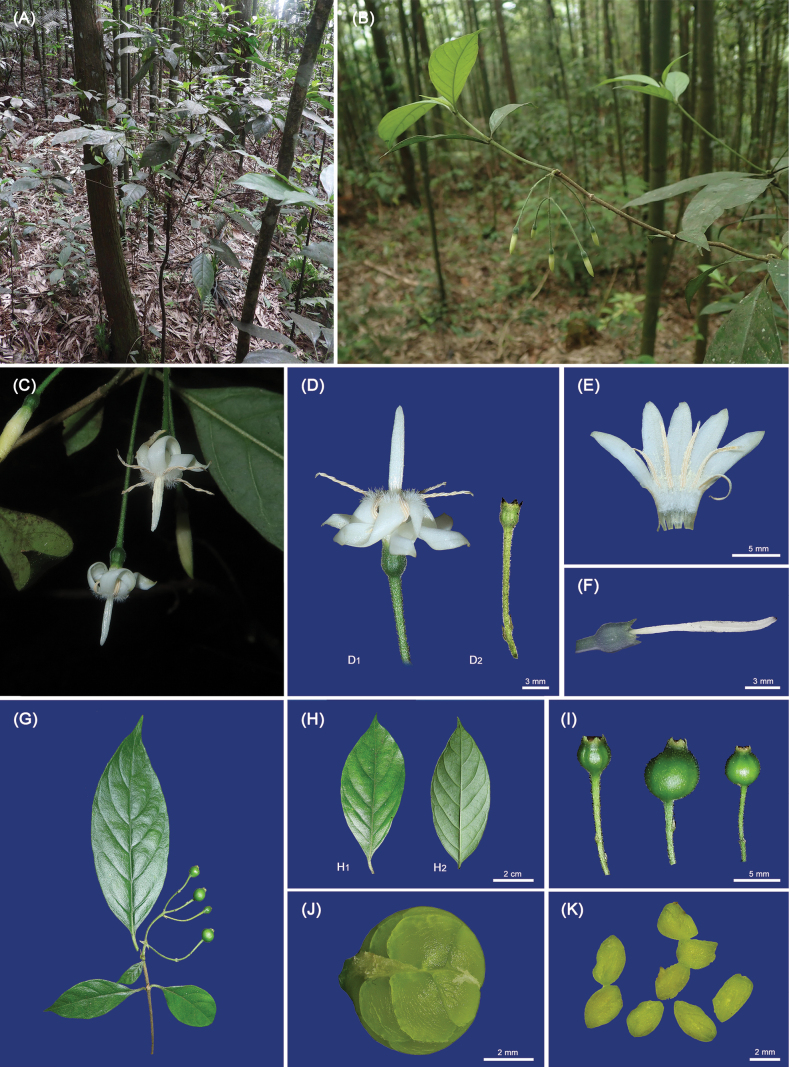
*Tarennapendula***A** habitat **B** flowering branch **C** pendulous cyme **D** flower (**D1**) and ovary and calyx (**D2**) **E** longitudinally opened corolla **F** ovary, calyx, style and stigma **G** fruiting branch **H** blade in adaxial view (**H1**) and in abaxial view (**H2**) **I** young fruits in different developmental stages **J** unripe seeds clustered together (after removal of fruit wall) **K** unripe seeds.

#### Phenology.

*Tarennapendula* has been observed to flower from May to July, and to fruit from July to January.

#### Distribution, habitat, and ecology.

**Figure 3.**
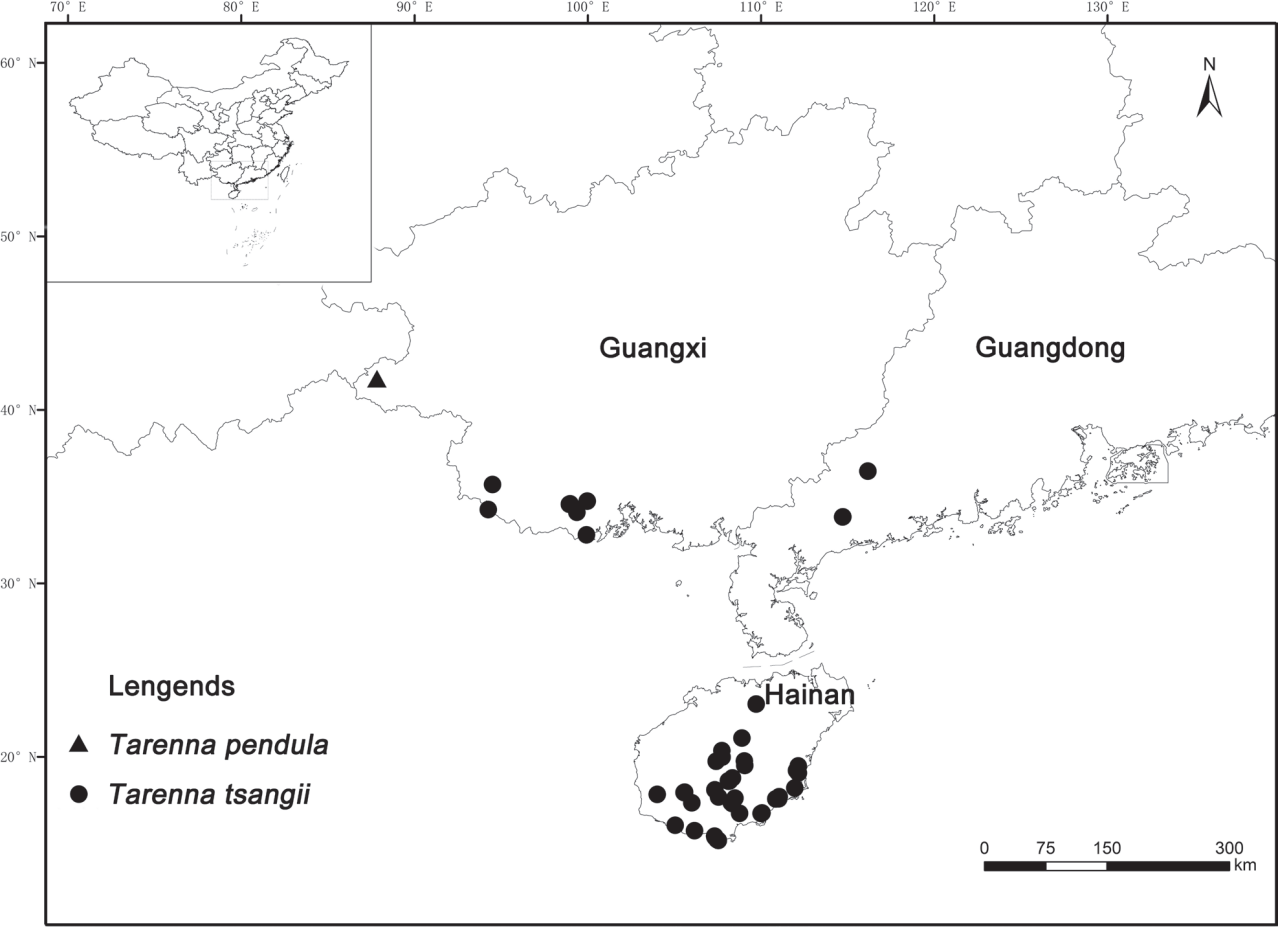
Distribution map of *Tarennapendula* (triangle) and *Tarennatsangii* (dots).

*Tarennapendula* grows in southern subtropical evergreen broadleaf forests, particularly in mixed bamboo understory (*Indosasasinica* C.D.Chu & C.S.Chao), at 1380 m a.s.l. The species is only known from this single locality in Napo County, Guangxi Autonomous Region, China (Fig. 3).

#### Conservation status.

*Tarennapendula* is only known from Baxiong Mountain, Defu Nature Reserve, Napo County, Guangxi, China with about 250 individuals recorded to date. Currently the species is only known from a single location. This location is inside a well-protected nature reserve, and as such, the species does not qualify for any threat condition. However, during fieldwork we noticed that the understory of the evergreen broadleaf forests in which *T.pendula* grows is being colonized by luxuriant bamboo, which may constitute a danger to the new species. A second possible threatening factor for this apparently rare and narrow-ranged species is a limited capacity of natural regeneration. The conservation status for *Tarennapendula* thus needs further study. We therefore assess the species as Data Deficient.

#### Etymology.

The specific epithet is derived from the pendulous inflorescence, which differs from several other *Tarenna* spp. in the area.

#### Vernacular name.

*Chuixiuwukoushu* in Mandarin Chinese, which translates to ‘pendulous *Tarenna*’.

#### Similar species.

*Tarennapendula* is similar to *T.tsangii* but is easily distinguished by its small form. Further diagnostic morphological characters of the new species and related species are presented in Table 1. Character information about relevant species is retrieved from Flora of China (Chen and Taylor 2011).

**Table 1.** Morphological comparison of *Tarennapendula* and *T.tsangii*.

	*T.pendula*	*T.tsangii*
Habit	Small shrubs, 1–2 m tall	Shrubs to trees, 1–6 m tall
Leaves	Asymmetrical, oblong–obovate or oblanceolate, 4–17 × 1.5–4 cm	Symmetrical, oblong–obovate or lanceolate, 5–26 × 1.5–7 cm
Stipules	Triangular, 3–5 mm, acuminate or apiculate when longer	Triangular, 4–5 mm, acuminate or apiculate
Blades	Adaxially with adnate short hairs	Adaxially glabrous
Pedicels	15–35 mm long	4–7 mm long
Cymes	Pendulous, with 3–5 or sometimes 9 flowers	Erect, with more than 10 flowers
Corolla	Corolla 1.5–1.9 cm long, tube ca. 5 mm long	Corolla 1.8–1.9 cm long, tube ca. 8–9 mm long
Styles	1–1.2 cm long	ca. 2 cm long
Ovary	Multiple ovules per locule	Two ovules per locule
Fruits	Sparsely pubescent	Glabrous
Seeds per fruit	13–19 seeds	4 seeds

It is challenging to distinguish different species based on morphological characters alone. However, after carefully scrutinizing the morphological characters of the new species and related species, we found that the new species is easily recognized by its pendulous cymose inflorescences and large number of seeds per fruit. Although *T.tsangii* has a much larger distribution range than *T.pendula*, their areas do not overlap. Because the distribution range of *T.pendula* is immediately adjacent to that of *T.tsangii*, we hypothesize that these two species may be allopatric. Given the complicated evolutionary relationship among *Tarenna* species, future studies should investigate the plant diversity and phylogeny of this genus in greater detail.

## Supplementary Material

XML Treatment for Tarenna pendula

## ﻿Additional information

### Author ORCIDs

Yong-Hua Qin https://orcid.org/0000-0002-9833-4193

Rainer W. Bussmann https://orcid.org/0000-0002-3524-5273

Zhi-Rong Liu https://orcid.org/0009-0001-5171-5693

Man Li https://orcid.org/0009-0006-6985-4882

Chang-Ying Xia https://orcid.org/0000-0002-5071-0145

Wang-Hui Wu https://orcid.org/0009-0000-1565-2376

Sheng-Xiang Yu https://orcid.org/0000-0002-5823-2870

### Data availability

All of the data that support the findings of this study are available in the main text.
